# Hantavirus reservoir hosts associated with peridomestic habitats in Argentina.

**DOI:** 10.3201/eid0506.990608

**Published:** 1999

**Authors:** G Calderón, N Pini, J Bolpe, S Levis, J Mills, E Segura, N Guthmann, G Cantoni, J Becker, A Fonollat, C Ripoll, M Bortman, R Benedetti, D Enria

**Affiliations:** Instituto Nacional de Enfermedades Virales Humanas "Dr. Julio I. Maiztegui", Buenos Aires, Argentina.

## Abstract

Five species of sigmodontine rodents have been identified in Argentina as the putative reservoirs of six circulating hantavirus genotypes. Two species of Oligoryzomys are associated with the genotypes causing hantavirus pulmonary syndrome, Oligoryzomys flavescens for Lechiguanas and O. longicaudatus for Andes and Oran genotypes. Reports of human cases of hantavirus pulmonary syndrome prompted rodent trapping (2,299 rodents of 32 species during 27,780 trap nights) at potential exposure sites in three disease-endemic areas. Antibody reactive to Sin Nombre virus was found in six species, including the known hantavirus reservoir species. Risk for peridomestic exposure to host species that carry recognized human pathogens was high in all three major disease-endemic areas.


### Hantavirus Reservoir Hosts Associated with Peridomestic Habitats in Argentina

**Gladys Calderón,* Noemí Pini,* Jorge Bolpe,† Silvana Levis,* James Mills,‡ Elsa Segura,* Nadia Guthmann,§ Gustavo Cantoni,¶ José Becker,* Ana Fonollat,# Carlos Ripoll,** Marcelo Bortman,†† Rosendo Benedetti,‡‡ Marta Sabattini,§§ and Delia Enria***

*Instituto Nacional de Enfermedades Virales Humanas "Dr. Julio I. Maiztegui," ANLIS, "Dr. Carlos G. Malbrán," Pergamino, Buenos Aires, Argentina; †Departamento de Zoonosis Rurales de Azul, Azul, Buenos Aires, Argentina; ‡Centers for Disease Control and Prevention, Atlanta, Georgia, USA; §Universidad Nacional del Comahue, S.C. de Bariloche, Rio Negro, Argentina; ¶Consejo Provincial de Salud Pública, Rio Negro, Argentina; #Fundación Lillo, San Miguel de Tucumán, Argentina; **Departamento de Chagas y Patologías Regionales, San Salvador de Jujuy, Argentina; ††Subsecretaría de Salud, Neuquén, Argentina; ‡‡Zona Sanitaria Noroeste, Esquel, Chubut, Argentina; and the Hantavirus Study Group[1]

Five species of sigmodontine rodents have been identified in Argentina as the putative reservoirs of six circulating hantavirus genotypes. Two species of Oligoryzomys are associated with the genotypes causing hantavirus pulmonary syndrome, *Oligoryzomys flavescens* for Lechiguanas and *O. longicaudatus* for Andes and Oran genotypes. Reports of human cases of hantavirus pulmonary syndrome prompted rodent trapping (2,299 rodents of 32 species during 27,780 trap nights) at potential exposure sites in three disease-endemic areas. Antibody reactive to Sin Nombre virus was found in six species, including the known hantavirus reservoir species. Risk for peridomestic exposure to host species that carry recognized human pathogens was high in all three major disease-endemic areas.

Hantaviruses, a genus in the family Bunyaviridae, are rodentborne pathogens producing chronic persistent infections in their reservoir hosts. Although the exact mechanism of transmission from rodents to humans is unknown, strong evidence suggests that these viruses are infectious by aerosols. Inhalation of aerosolized virus from rodent excreta is thought to be the main route of transmission to humans (1).

Although hantaviruses have been reported in the Americas since the 1980s (2,3), before 1993 human illnesses caused by hantaviruses, grouped under the name of hemorrhagic fever with renal syndrome, were thought to be limited to Europe and Asia. After hantavirus pulmonary syndrome (HPS) was described as a clinical form of hantavirus illnesses in the New World, outbreaks of HPS as well as isolated cases were recognized in many parts of the Americas. In Argentina, where cases of HPS were identified retrospectively as early as the 1980s (4), three geographically and ecologically distinct HPS-endemic areas have been recognized (5): the northern zone, a subtropical area bordering the Bermejo River; the central zone, a region of humid plains and temperate climate; and the southern zone, a cold, forested region bordering the Andean range (Figure).

The common rodents in populated areas of Argentina belong to two groups of the family Muridae. The most common rodents in natural, as well as disturbed habitats outside urban and peridomestic areas, are numerous species of the

Address for correspondence: Gladys Calderón, Instituto Nacional de Enfermedades Virales Humanas "Dr. Julio I. Maiztegui," Monteagudo 2510, (2700) Pergamino, Buenos Aires, Argentina; fax: 54-2477-433045; e-mail: gladys@inevh.sld.ar.

[1]Eduardo Herrera and Edmundo Larrieu, Consejo Provincial de Salud Pública, Rio Negro, Argentina; María Cacace, Hospital San Vicente de Paul, Orán, Salta, Argentina; Roberto Gonzalo, Ricardo Fernandez, Gustavo Martinez, and Alberto Suzzi, Zona Sanitaria Noroeste, Esquel, Chubut, Argentina.

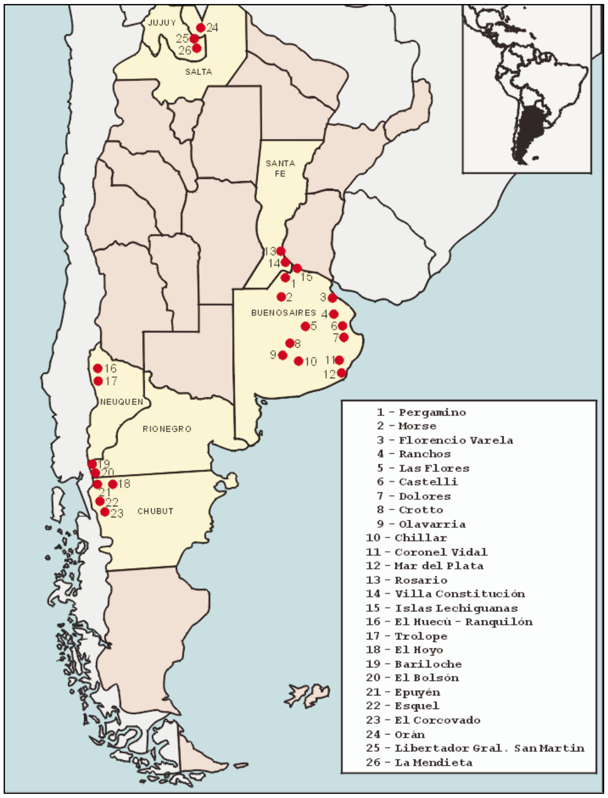

Figure. Sites of rodent trapping and human cases in three hantavirus pulmonary syndrome-endemic zones in Argentina.

Murid subfamily, Sigmodontinae (the New World rats and mice) (6). All hantaviruses known to cause HPS are associated with sigmodontine rodents. The common rodents in towns, cities, and peridomestic (in and around homes) environments are three introduced species of the subfamily Murinae: *Rattus rattus* (black rat), *R. norvegicus* (Norway rat), and *Mus musculus* (house mouse) (6).

In South America, hantaviruses are associated with several species of indigenous sigmodontine rodents. In Argentina, seven viral genotypes have been described: Bermejo and Oran in the northern zone; Lechiguanas, Hu39694, Maciel, and Pergamino in the central zone; and Andes in the southern zone (7,8). Andes, Lechiguanas, Hu39694, and Oran have been associated with human disease, and the putative reservoirs of three of these genotypes are two species of *Oligoryzomys*: *O. longicaudatus* from southern Argentina for Andes, *O. longicaudatus* from northern Argentina for Oran, and *O. flavescens* for Lechiguanas. *O. longicaudatus* (reservoir of Oran and Andes genotypes) may represent two species (8). The putative reservoir for the Bermejo genotype, not yet associated with human disease, is reported to be *O. chacoensis*. The reservoir for Hu39694 is unknown, although its close genetic similarity to Andes, Oran, and Bermejo suggests that it may be another *Oligoryzomys* species from central Argentina. In the central zone, two genotypes not yet associated with HPS were identified from other sigmodontine species: Maciel, from *Necromys benefactus* (previously designated *Bolomys obscurus*), and Pergamino, from *Akodon azarae* (8).

Since 1996, follow-up investigations have been conducted when HPS cases in Argentina were confirmed. As of January 20, 1999, 210 cases of HPS had been confirmed in Argentina (Ministerio de Salud y Acción Social). This investigation includes rodent studies to identify areas in which HPS poses a high risk and to determine the spatial distribution of rodent reservoir populations in relation to the suspected sites of exposure for persons with HPS.

### Identification of HPS Cases and Study Areas

Confirmed cases of HPS were defined as having the following characteristics: 1) a compatible clinical illness and 2) laboratory evidence of acute hantavirus infection, such as a positive enzyme-linked immunosorbent assay (ELISA) hantavirus immunoglobulin (Ig) M or a fourfold rise in ELISA IgG; a positive reverse transcription-polymerase chain reaction (RT-PCR) for hantavirus RNA; or positive immunohistochemistry for hantavirus antigen. When an HPS case was confirmed, small mammals were trapped in collaboration with the local health authorities at the patient's home or work sites and neighboring areas (Figure).

### Selection and Classification of Potential Exposure Sites

The potential exposure sites were chosen by selecting all places where patients had been living or working or had visited during the 6 weeks before onset of symptoms. Rodents were trapped in all these sites, which were classified into six categories: domestic and peridomestic urban, domestic and peridomestic rural, other urban, and other rural. Peridomestic urban and rural categories were all sites in the immediate vicinity of homes or buildings, including yards, parks, driveways, adjoining lands, outbuildings, vegetable gardens, and fence lines. The peridomestic rural category includes ponds, natural or planted woodlots, weeds, sugar cane or plantain plantations, and corn stubble in the immediate vicinity of the house. All other trapping sites distant from the previously mentioned settings were considered other urban or other rural. Other urban includes sites from the outskirts of towns and natural and artificial corridors that could allow the access of sigmodontine rodents to urban areas, such as railroad rights-of-way and roadsides inside the perimeter of the town. In other rural sites rodents were captured in open fields, where the representative habitats of each area were sampled, including natural and modified land, such as cultivated areas and weeds.

### Small-Mammal Trapping and Processing

In the southern and central zones, rodents were trapped as soon as HPS case reports were received. In the remote northern zone, three expeditions were organized to trap rodents at sites frequented by six persons with HPS reported in previous months, and only rarely was trapping conducted inside houses. The three expeditions took place in July 1995, October 1996, and May 1998; rodents were trapped at 18 sampling sites.

From August 1994 to April 1998, 46 sampling sites were selected in the central zone. In the southern zone, we included 51 sampling sites from November 1996 to April 1998 (Table 1).

Each site was sampled with Sherman (8 x 9 x 23 cm) and Tomahawk (14 x 14 x 40 cm) live-capture traps. The number of traps depended on the area available for trap placement at each site. Animals were trapped and sampled according to established safety guidelines (9) and were anesthetized with Isoflurane (Abbott Laboratories) before blood was drawn from the retroorbital sinus. Carcasses were tentatively identified in the field and kept in a

Table 1. Relative density (as indicated by trap success[a]) for frequently captured rodent species in three hantavirus pulmonary syndrome-endemic zones in Argentina

| Zone/trap nights | Species[b] | Site type/no. | | | | | | |
|---|---|---|---|---|---|---|---|---|
| | | DU[c]/1 | PU/1 | DR/2 | PR/10 | OU/1 | OR/3 | All sites/18 |
| Northern | *Av* | 0 | 1.0 | 0 | 1.3 | 0.6 | 2.4 | 1.4 |
| | *Cc* | 0.7 | 1.0 | 0 | 0.9 | 1.2 | 1.3 | 1.0 |
| | *Och* | 0 | 1.0 | 0 | 0.8 | 0 | 0.1 | 0.7 |
| | *Ol* | 0 | 0 | 0 | 0.8 | 0 | 0.3 | 0.6 |
| | *As* | 0 | 0 | 2.6 | 0.5 | 0 | 0.1 | 0.5 |
| | *Mm* | 0.7 | 0 | 0 | <0.1 | 0 | 0.1 | 0.1 |
| | *Rr* | 0 | 0 | 41.0 | 0.2 | 1.2 | 0.1 | 0.5 |
| Trap nights | | 136 | 100 | 39 | 4,069 | 164 | 739 | 5,247 |
| | | DU/10 | PU/8 | DR/5 | PR/14 | OU/3 | OR/6 | All sites/46 |
| Central | *Aa* | 0 | 9.5 | 0 | 3.1 | 0 | 13.9 | 4.7 |
| | *Of* | 0 | 1.1 | 0.4 | 4.6 | 0 | 4.2 | 3.8 |
| | *Cm* | 0 | 0.4 | 0 | 0.5 | 0 | 3.5 | 0.8 |
| | *Cl* | 0 | 0.1 | 0.4 | 0.4 | 0 | 0.3 | 0.4 |
| | *Hb* | 0 | 0 | 0 | 0 | 0 | 1.7 | 0.2 |
| | *Mm* | 1.7 | 5.9 | 0.4 | 1.2 | 6.0 | 0.1 | 1.5 |
| | *Rr* | 0 | 0.1 | 0 | 0.1 | 0 | <0.1 | 0.1 |
| Trap nights | | 829 | 939 | 260 | 7,900 | 116 | 1,494 | 11,538 |
| | | DU/7 | PU/10 | DR/5 | PR/9 | OU/8 | OR/12 | All sites/51 |
| Southern | *Ol* | 1.6 | 0.2 | 0 | 6.1 | 0.8 | 5.4 | 3.2 |
| | *Al* | 0 | 0.5 | 0 | 0.9 | 0.8 | 3.5 | 1.6 |
| | *Ao* | 0 | <0.1 | 0 | 1.0 | <0.1 | 0.3 | 0.3 |
| | *Mm* | 0.4 | 0.5 | 0 | 0.9 | 0 | 0.2 | 0.3 |
| Trap nights | | 512 | 1,650 | 251 | 1,731 | 3,101 | 3,750 | 10,995 |

[a]Number of captures per 100 trap nights, where a trap night is one trap for one night.
[b]*Av, Akodon varius; Cc, Calomys callosus; Och, Oligoryzomys chacoensis; Ol, Oligoryzomys longicaudatus; As, Akodon spegazzinii; Mm, Mus musculus; Rr, Rattus rattus; Aa: Akodon azarae; Of, Oligoryzomys flavescens; Cm, Calomys musculinus; Cl, Calomys laucha; Hb, Holochilus brasiliensis; Al, Abrothrix longipilis; Ao, Abrothrix olivaceus.*
[c]DU, domestic urban; PU, peridomestic urban; DR, domestic rural; PR, peridomestic rural; OU, other urban; OR, other rural.

solution of 10% formalin for confirmation of identification at the Museum of Natural Sciences "Bernardino Rivadavia," Buenos Aires.

### Structure of Small-Mammal Communities

During 26,458 Sherman and 1,322 Tomahawk trap-nights, 2,299 small mammals belonging to two orders (Rodentia and Didelphimorphia) and three families (Muridae, Caviidae, and Didelphidae) were captured. These animals belonged to 32 species, with the murid subfamily Sigmodontinae representing 86.3% of the total sample.

The introduced murine rodents *R. rattus* and *M. musculus,* as well as *Cavia aperea* (Caviidae), were captured in all three areas. Sigmodontine rodents were represented by different species in the three regions.

### Distribution of Species by Site of Capture

In all three regions, *M. musculus* was found in domestic urban sites (Table 1). In two of the three areas, we also observed rodents inside urban homes; this is the first documented occurrence of sigmodontine species entering homes in Argentina.

We also found sigmodontine rodents inside rural homes: one *Calomys laucha* and one *O. flavescens* in the central zone and one *Akodon spegazzinii* in the northern zone. Sigmodontine rodents, including the reservoirs for Lechiguanas and Andes viruses, were also captured in the peridomestic urban sites, especially in the central and southern zones. In peridomestic rural habitats next to open fields, captures of sigmodontines were expected. The trap success values for hantavirus reservoir species in peridomestic rural sites were similar or higher than those in open fields represented by other rural sites. The relative proportion of rodent species among site categories includes all species antibody positive and the species that were numerically dominant but antibody negative in each zone. The category "others" includes species that were less representative in each zone; the high values observed in PU and OU sites in the northern zone were due to the low number of

Table 2. Relative proportion[a] of rodent species in each site category, by site

| | | Site type/total no. captured | | | | | | p[c] | | |
|---|---|---|---|---|---|---|---|---|---|---|
| Zone | Species[b] | DU/2 | PU/5 | DR/18 | PR/227 | OU/10 | OR/58 | PRvsOR | | |
| Northern | *Av* | 0 | 20.0 | 0 | 23.8 | 10.0 | 31.0 | NS | | |
| | *Cc* | 50.0 | 20.0 | 0 | 17.2 | 20.0 | 17.2 | NS | | |
| | *Och* | 0 | 20.0 | 0 | 14.5 | 0 | 1.7 | * | | |
| | *Ol* | 0 | 0 | 0 | 14.1 | 0 | 3.4 | * | | |
| | *As* | 0 | 0 | 5.6 | 9.7 | 0 | 1.7 | NS | | |
| | *Mm* | 50.0 | 0 | 0 | 1.3 | 0 | 1.7 | NS | | |
| | *Rr* | 0 | 0 | 88.9 | 4.0 | 20.0 | 1.7 | NS | | |
| | Others | 0 | 40.0 | 5.6 | 15.4 | 50.0 | 41.4 | * | | |
| | | DU/14 | PU/162 | DR/3 | PR/805 | OU/7 | OR/389 | PUvsPR | PRvsOR | PUvsOR |
| Central | *Aa* | 0 | 54.9 | 0 | 30.3 | 0 | 53.2 | * | * | NS |
| | *Of* | 0 | 6.2 | 33.3 | 45.3 | 0 | 16.2 | * | * | * |
| | *Cm* | 0 | 2.5 | 0 | 5.1 | 0 | 13.6 | NS | * | * |
| | *Cl* | 0 | 0.6 | 33.3 | 4.3 | 0 | 1.3 | * | * | NS |
| | *Hb* | 0 | 0 | 0 | 0 | 0 | 6.4 | ND | * | * |
| | *Mm* | 100 | 33.9 | 33.3 | 11.5 | 100 | 0.5 | * | * | * |
| | *Rr* | 0 | 0.6 | 0 | 1.1 | 0 | 0.3 | NS | NS | NS |
| | Others | 0 | 1.2 | 0 | 2.2 | 0 | 8.5 | NS | * | * |
| | | DU/10 | PU/21 | DR/0 | PR/161 | OU/55 | OR/355 | PRvsOU | PRvsOR | OUvsOR |
| Southern | *Ol* | 80.0 | 14.3 | 0 | 65.8 | 47.3 | 57.5 | * | NS | NS |
| | *Al* | 0 | 38.1 | 0 | 9.3 | 45.4 | 36.6 | * | * | NS |
| | *Ao* | 0 | 4.8 | 0 | 10.6 | 1.8 | 3.1 | NS | * | NS |
| | *Mm* | 20.0 | 38.1 | 0 | 9.3 | 0 | 2.0 | * | * | NS |
| | Others | 0 | 4.8 | 0 | 5.0 | 5.4 | 0.8 | NS | * | * |

[a]Calculated as the percentage of total captures in a given site category represented by each species.
[b]*Av, Akodon varius; Cc, Calomys callosus; Och, Oligoryzomys chacoensis; Ol, Oligoryzomys longicaudatus; As, Akodon spegazzinii; Mm, Mus musculus; Rr, Rattus rattus: Aa, Akodon azarae; Of, Oligoryzomys flavescens; Cm, Calomys musculinus; Cl, Calomys laucha; Hb, Holochilus brasiliensis; Al, Abrothrix longipilis; Ao, Abrothrix olivaceus.*
[c]Chi-square test for comparison of two proportions in two independent samples. Epi Info version 6.04.
*p <0.05; NS, p >0.05; ND, not done. Comparisons were made and are shown only for cases where sample size was sufficient for statistical comparisons.

captures and in OR to the high diversity of species captured (Table 2). The relative proportion was compared by chi-square test with Epi Info version 6.04. Only site categories with ≥30 captures could be tested. An increase in the relative proportion of *O. flavescens* (host of the genotype Lechiguanas, associated with human disease) in the central zone and *O. longicaudatus* (putative reservoir of the genotype Orán, also associated with human disease) in the northern zone was seen in peridomestic rural settings in comparison with other rural. *O. longicaudatus* (proposed reservoir for Andes virus) was captured in similar relative proportions in both peridomestic and other rural sites. In all cases, these findings emphasize the risk linked to peridomestic settings.

### Hantavirus Infection in Rodents

We tested 2,159 (93.9%) rodents in IgG ELISA by using Sin Nombre virus antigen (CDC, SPR293). We used a recombinant nucleocapsid protein as antigen applied to the solid phase of a microtiter plate. Hantavirus-specific IgG in test samples of rodent whole blood was allowed to bind to the antigen. A mixture of two conjugates (anti-*Peromyscus leucopus* and anti-*Rattus norvegicus*, Kirkegaard and Perry) was used to detect immune globulins from various murid rodent phyla. This was followed by 2,2'-azino-di(3-ethybenthiazonline sulfonate) substrate (Kirkegaard and Perry Laboratories, Inc.) and read with a Bio-Tek Microplate autoreader at 405 and 450 nm. A titer ≥1:400 was considered positive (10).

Of 330 rodents tested in the north, 5 (1.5%) were positive (Table 3). In the central zone, we found 35 (2.6%) positives among 1,326 rodents, associated with eight HPS cases. In the south, 27 (5.4%) of 503 rodents tested had positive results. In the northern zone, the presence of infected *O. longicaudatus* was associated with HPS cases in peridomestic rural habitats. The importance of detecting infected *O. chacoensis* and *Akodon varius* associated with an HPS case in peridomestic urban and rural sites cannot be assessed until data on the viral genotypes of the rodents and the case patients are available.

In the central zone, apart from *O. flavescens,* already shown to be associated with HPS,

Table 3. Antibody distribution in rodents, by province, species, and site category

| Site zone[a] | Province | Immunoglobulin G antibody[b] species (pos/tested) (%) | Site category[c] |
|---|---|---|---|
| Northern | Jujuy | *Oligoryzomys chacoensis* 1/12 (8.3) | PU |
| | Salta | *O. chacoensis* 1/27 (3.7) | PR |
| | | *Akodon varius* 1/26 (3.8) | PR |
| | | *O. longicaudatus* 2/26 (7.7) | PR |
| | | Other species 0/239 (0.0) | |
| Central | Buenos Aires | *O. flavescens* 8/170 (4.7) | PR-OR |
| | | *A. azarae* 15/549 (2.7) | PU-PR-OR |
| | Entre Rios | *O. flavescens* 11/243 (4.5) | PR |
| | | *H. brasiliensis* 1/30 (3.3) | PR |
| | | Other species 0/334 (0.0) | |
| Southern | Rio Negro | *O. longicaudatus* 18/195 (9.2) | PR-OR |
| | Chubut | *O. longicaudatus* 5/40 (12.5) | PR-OR |
| | Neuquén | *O. longicaudatus* 4/88 (4.5) | PR |
| | | Other species 0/180 (0.0) | |

[a]Animals tested and found negative, by zone and species.

Northern: *Akodon varius* (48), *A. boliviensis* (1), *A. albiventer* (1), *A. spegazzinii* (26), *Akodon* sp. (10), *Calomys callosus* (53), *C. laucha* (2), *Calomys* sp. (1), *Cavia aperes* (3), *Eligmodontia moreni* (6), *Galea musteloides* (2), *Graomys griseoflavus* (2), *Holochilus brasiliensis* (1), *H. chacarius* (4), *Mus musculus* (6), *Oligoryzomys flavescens* (1), *O. longicaudatus* (8), *Oligoryzomys* sp. (10), *Oxymycterus paramensis* (5), *Phillotys osilae* (2), *Rattus rattus* (28), *Rattus* sp. (2), *Thylamys elegans* (5), and unidentified (13). Total 240.

Central: *Calomys musculinus* (94), *C. laucha* (40), *Necromys benefactus* (2), *Oxymicterus rufus* (9), *Mus musculus* (157), *Rattus rattus* (8), *R. norvegicus* (1), *Cavia aperes* (1), *Monodelphia dimidiata* (3), and unidentified (19). Total 334.

Southern: *Abrotrix longipilis* (138), *A. oliveceus* (25), *Mus musculus* (12), *Rattus rattus* (1), *Thylamys elegans* (1), and unidentified (3). Total 180.

[b]Enzyme-linked immunosorbent assay using Sin Nombre virus antigen.

[c]PU, peridomestic urban; PR, peridomestic rural; OR, other rural. Seropositive animals were found only in these three site categories.

another species found infected was *A. azarae*, the putative reservoir of the Pergamino genotype, which has not yet been associated with human disease. Spatial and temporal association between an HPS case and an infected *A. azarae* does not confirm this species as the source of infection. Further genetic studies are under way to determine if Pergamino virus was responsible for the HPS cases.

In the southern zone, human cases were associated with *O. longicaudatus* captured in peridomestic and other rural settings (Table 3). In the three zones, in all other site categories, no seropositive animals were found. Nevertheless, because of small sample sizes, any conclusions concerning lack of infection in these site categories are tentative.

### Conclusions

Infected hantavirus reservoir hosts (as evidenced by antibody positivity) were found within peridomestic environments in all three HPS-endemic zones in Argentina. Reservoir species were captured inside urban houses in two of the three endemic zones. Although host species were not captured in homes in the northern zone, sampling was not sufficient to exclude the possibility that they enter homes occasionally.

The presence of hantavirus reservoir species in peridomestic environments indicates risk for human inhabitants. The primary measure for reducing the risk is preventing access of rodents to homes (11). The efficacy of proposed and currently used exclusion methods in Argentina needs to be evaluated (12).

Sigmodontine rodents, including known hantavirus reservoir species, were frequently captured in the rural and small-town peridomestic environments we studied. At many of the case sites, the level of hygiene was suboptimal. The widespread presence of such conditions underscores the importance of local habitat management to prevent wild (sigmodontine) rodents from entering domestic areas in towns, villages, and urban centers and of health education for the local population to reduce the risk for hantavirus infection.

### Acknowledgments

We thank Horacio Lopez, Diego Olivera, Mario Palmigiano, Felix May, Oscar Gallicchio, Anibal Hirsch, Horacio Larraburu, Mariana Lozada, Pablo Sandoval, Federico Bianconi, Mario Diaz, Malcom Elder, Carmelo Saavedra, and Omar Fuentes for the rodent trapping effort; T. Ksiazek for providing antigen for the enzyme-linked immunosorbent assay; and Marta Piantanida, Elio Massoia, and Jaime Polop for identification of rodent specimens.

This work was supported in part by grant N° US 1181199 from the World Health Organization and by Administración Nacional de Laboratorios e Institutos de Salud (ANLIS) "Dr. Carlos G. Malbrán," Ministerio de Salud Pública de la Nación.

Dr. Calderón is chief of the Quality Control Division at the Instituto Nacional de Enfermedades Virales Humanas "Dr. Julio I. Maiztegui." Her research interests include rodentborne diseases, specifically those caused by arenaviruses and hantaviruses.

### References

1. McKee KT Jr, LeDuc JW, Peters CJ. Hantaviruses. In: Belshe RB, editor. Textbook of human virology. St. Louis (MO): Mosby Year Book; 1991.
2. LeDuc JW, Smith GA, Childs JE, Pinheiro FP, Maiztegui JI, Niklasson B et al. Global survey of antibody to Hantaan-related viruses among peridomestic rodents. Bull WHO 1986;64:139-44.
3. Weissenbacher MC, Cura E, Segura E, Hortal M, Back LJ, Yong K, et al. Serological evidence of human hantavirus infection in Argentina, Bolivia and Uruguay. Medicina (Buenos Aires) 1996;56:17-22.
4. Parisi M, Enria D, Pini N, Sabattini MS. Detección retrospectiva de infecciones clínicas por hantavirus en la Argentina. Medicina (Buenos Aires) 1996;56:1-13.
5. Levis SC, Briggiler AM, Cacase M, Peters CJ, Ksiazek TG, Cortés J, et al. Emergence of hantavirus pulmonary syndrome in Argentina. Proceedings from the 44th Annual Meeting; 1995 Nov 17-21; San Antonio, Texas. American Society of Tropical Medicine and Hygiene; 1995. p. 233.
6. Redford K, Eisenberg J. Mammals of the neotropics. The southern cone. Vol 2. The University of Chicago Press; 1992.
7. Lopez N, Padula R, Rossi C, Lázaro ME, Franze-Fernandez MT. Genetic identification of a new hantavirus causing severe pulmonary syndrome in Argentina. Virology 1996;220:223-6.
8. Levis S, Morzunov S, Rowe J, Enria D, Pini N, Calderón G, et al. Genetic diversity and epidemiology of hantaviruses in Argentina. J Infect Dis 1998;177:529-38.
9. Mills J, Childs J, Ksiazek T, Peters CJ, Velleca W. Métodos para trampeo y muestreo de pequeños mamíferos para estudios virológicos. Washington: Organización Panamericana de la Salud; 1998. Report #OPS/HPS/HCT98.104.
10. Ksiazek T, Peters CJ, Rollin PE, Zaki S, Nichol S, Spiropoulou C, et al. Identification of a new North American hantavirus that causes acute pulmonary insufficiency. Am J Trop Med Hyg 1995;52:117-23.
11. Centers for Diseases Control and Prevention. Hantavirus infection-southwestern United States: interim recommendations for risk reduction. MMWR Morb Mortal Wkly 1993;42(RR-11):1-13.
12. Glass G, Johnson J, Hodenbach G, Disalvo C, Peters CJ, Childs J, et al. Experimental evaluation of rodent exclusion methods to reduce hantavirus transmission to humans in rural housing. Am J Trop Med Hyg 1997;56:359-64.
